# How Does It Feel to Be Online? Psychotherapists’ Self-Perceptions in Telepsychotherapy Sessions During the COVID-19 Pandemic in Italy

**DOI:** 10.3389/fpsyg.2021.726864

**Published:** 2021-09-03

**Authors:** Elisa Mancinelli, Emanuela S. Gritti, Arianna Schiano Lomoriello, Silvia Salcuni, Vittorio Lingiardi, Tommaso Boldrini

**Affiliations:** ^1^Department of Developmental Psychology and Socialization, University of Padova, Padova, Italy; ^2^Section for Cognitive Systems, DTU Compute, Technical University of Denmark, Lyngby, Denmark; ^3^Department of Dynamic and Clinic Psychology, Faculty of Medicine and Psychology, Sapienza University of Rome, Rome, Italy

**Keywords:** psychotherapists, telepsychotherapy, remote psychotherapy, online psychotherapy, COVID-19, self-perception, semantic differential method

## Abstract

**Aims:** The COVID-19 pandemic and consequent extreme restrictions imposed by governments across the world forced psychotherapists to abruptly change their working modality. The first aim of the current study was to assess psychotherapists’ self-perceptions (i.e., affective and cognitive perceptions about their self and their self in relation to clients) when providing telepsychotherapy during the first peak of the COVID-19 pandemic in Italy. The second aim was to explore the associations between psychotherapists’ self-perceptions, characteristics, and clinical practices.

**Method:** An online survey was administered to 281 Italian licensed psychotherapists (*M_age_*=45.15; *SD*=10.2; 83.6% female) between April 5 and May 10, 2020. The survey comprised *ad-hoc* questions that were designed to collect sociodemographic details and information related to working practices. Moreover, a semantic differential (SD) scale was developed to assess psychotherapists’ self-perceptions, and a factor analysis was performed from the SD items.

**Results:** The SD scale showed an overall trend of positive psychotherapist self-perception during telepsychotherapy, despite reports of greater fatigue and directive and talkative behavior during sessions. Four SD factors accounted for 45% of the variance: “Affective Availability,” “Attitude Predisposition,” “Well-being,” and “Interventionism.” Scores on the first three factors were indicative of psychotherapists’ Positive vs. Negative self-perception. A comparison of the Positive and Negative attitudinal profiles using the chi-squared test with Yates’s correction and a Monte Carlo simulation found that psychotherapists with a Positive profile reported greater satisfaction with the telematic modality and were more likely to perceive that their clients were able to maintain privacy during sessions.

**Conclusion:** The results suggest that Italian psychotherapists have been able to promptly adapt to the imposed telematic modality during the COVID-19 pandemic. However, they may have attempted to compensate for their physical distance from clients by intervening more during sessions. These findings may support psychotherapists who are currently practicing and inform future practitioners who are considering the use of telematic treatment as a routine component of psychotherapeutic care.

## Introduction

The rapid escalation of the Sars-Cov-2 outbreak to a pandemic in March 2020, following the first documented infection in mainland China in December 2019 (Li et al., 2020), triggered multiple psychological sequelae in the society at large. These effects were ubiquitous across global regions and population ages, with specific manifestations for particular social groups, such as the general population (e.g., Cao et al., 2020; Boldrini et al., 2021; Castellini et al., 2021), frontline workers (Nowicki et al., 2020; Salazar et al., 2020), and COVID-19 patients (e.g., Jiang et al., 2021). For the general population, the pandemic added an additional psychological burden on top of preexisting mental healthcare needs. This, in conjunction with the limitations to physical contact and social proximity enforced by governmental authorities to contain the spread of infection, meant that the online delivery of mental care (e.g., telepsychotherapy) became an urgent substitute for face-to-face treatment (Inchausti et al., 2020). In Italy, the National Council of Psychologists invited psychologists and psychotherapists to provide their professional services via digital devices during the pandemic. This suggestion aimed to guarantee the continuation of previously active therapeutic treatments and to ensure mental health support for diseases linked to pandemic and quarantine, as far as possible.

Despite the evident need for this form of remote psychotherapeutic intervention, moderate skepticism regarding alternatives to in-person treatment exists from persons inside and outside the clinical profession (see Poletti et al., 2020, for a review). Among many clinicians, telepsychotherapy was viewed as a less authentic and less effective form of psychotherapy (see Weinberg, 2020, for a review); and among the general public, there was limited knowledge on this treatment option and concerns about a possible drop in treatment quality relative to in-person therapy (Apolinário-Hagen et al., 2017).

Despite this criticism, a growing body of research has provided neutral or favorable views of telepsychotherapy (e.g., van der Vaart et al., 2014), documenting the effectiveness of this mode of treatment as equivalent to in-person treatment, especially during large-scale health emergencies (Backhaus et al., 2012). During the current pandemic, in response to the need for flexible and prompt clinical care (Duan and Zhu, 2020), online interventions have become widely used (American Psychological Association, 2020). Research has shown that these interventions are registering good success for a variety of social groups and clinical conditions (Inchausti et al., 2020).

Psychotherapists who previously delivered their service online prior to the COVID-19 pandemic might now be experiencing the advantages and challenges of this modality differently, due to the abrupt change and personal impact of the global emergency. However, it is most likely that the transition to telepsychotherapy has been particularly challenging for clinicians with no previous experience of telepsychotherapy (Boldrini et al., 2020) and/or a strong preference for in-person treatment (Reay et al., 2020). Indeed, clinicians delivering telepsychotherapy have reported concerns about their ability to be authentically and emotionally connected to their clients, as well as their ability to be as effective as they would otherwise be in a traditional therapy setting (Aafjes-van Doorn et al., 2020; Békés and van Doorn, 2020; Messina and Löffler-Stastka, 2021).

The literature depicts a mixed and heterogeneous scenario in terms of psychotherapists’ emotional experiences and general satisfaction with telepsychotherapy during the pandemic. In a sample of 141 therapists from the United States interviewed at the end of March 2020, for instance, clinicians reported that they felt less competent and less confident during online compared to in-person sessions (Aafjes-van Doorn et al., 2020). However, despite their concern that clients might perceive them as more aloof during online sessions, they also reported fairly good therapeutic relationship and alliance. These results are aligned with those of a study on a similar cohort of therapists during the same stage of the pandemic, according to which only 21.4% of the clinicians expressed problems communicating empathy and feelings toward their clients online (Békés and van Doorn, 2020). An Israeli study in a couples’ therapy setting and involving slightly older clinicians (*M*_age_=50.5years, *SD*=9.5) and different professional roles (i.e., 28% psychologists and the rest other designations, such as social workers) produced encouraging results (Machluf et al., 2021), especially with respect to comparisons with prior face-to-face treatment. The main reported challenges were therapists’ concerns about their ability to create and maintain a solid therapeutic alliance with each partner, to manage conflict, and to prevent dropout.

Research on telepsychotherapy before and during the COVID-19 crisis has shown that both clients’ and clinicians’ expectations toward the online treatment modality significantly affect its efficacy (Reese et al., 2016; Tonn et al., 2017). Thus, it became essential to investigate psychotherapists’ attitudes toward telepsychotherapy and their self-perceptions during sessions. Studies on the effects of the pandemic on psychotherapists’ online working, in terms of affective reactions and perceived differences from previous working styles, are quantitatively limited (e.g., Feijt et al., 2020). Furthermore, most research on this subject has focused on United States or Canadian clinicians or involved small samples (Aafjes-van Doorn et al., 2020; Feijt et al., 2020; Messina and Löffler-Stastka, 2021). Therapists’ affective states and self-perceptions during online treatment are fundamental in determining the quality of the mental care; indeed, it is likely that these factors play a more relevant role than technical and communication challenges in shaping therapists’ attitudes to telepsychotherapy (Békés and van Doorn, 2020).

The first aim of the current study is to quantitatively explore and assess psychotherapists’ self-perceptions (i.e., affective and cognitive perceptions about their self and their self in relation to clients) when providing telepsychotherapy during the first peak of the COVID-19 pandemic in Italy. The second aim is to explore the association between psychotherapists’ subjective perceptions during online sessions and their characteristics (i.e., theoretical orientation, working practice, previous experience with telepsychotherapy, and personal beliefs about telepsychotherapy) and clinical practices (i.e., proportion of clients treated via telephone or video call, dropout rate due to COVID-19, and perception of clients’ ability to maintain privacy during sessions).

## Materials and Methods

### Procedure

The study was conducted in Italy during the first COVID-19 national lockdown (beginning March 9, 2020), which lasted approximately 10weeks. Data collection began approximately 4weeks after the start of the lockdown (April 4, 2020) and continued until May 10, 2020. Participants completed an online survey that was developed *ad-hoc* as part of a broader research project (see Boldrini et al., 2020). Recruitment was performed using the snowballing method. Participation was voluntary, and all participants were informed that the data were collected anonymously and would only be analyzed in aggregate. Moreover, they were informed that the collected data would be protected under the EU GDPR (2016, pd. 196/03). The study was conducted in compliance with the Declaration of Helsinki.

### Measures

The online survey comprised 45 items. In addition to collecting sociodemographic information, the questionnaire also investigated psychotherapists’ clinical practice (i.e., work context, setting, and experience with telepsychotherapy), theoretical orientation, beliefs about their theoretical compatibility with the telematic setting, personal satisfaction with telepsychotherapy, proportion of clients treated via telephone or video call, client dropout due to the impossibility of in-person psychotherapy, and perception of clients’ ability to maintain privacy during telepsychotherapy sessions.

Moreover, the survey included items investigating psychotherapists’ subjective experiences, referring to their affective and cognitive perceptions of their self and their self in relation to clients during the lockdown period. Specifically, participants were asked to rate their self-perception (*“During the telematic sessions in this time of emergency, I feel I am:”*) on a scalar continuum between opposite or polar pairs of adjectives, following the sematic differential (SD) method. The SD method operationalizes qualitative information, measuring respondents’ affective, and attitudinal states based on the attributional meaning given to items (Osgood et al., 1957). The methodology is well established as a reliable and effective way to measure attitudes, preferences, and perceptions resulting from real-life experience (e.g., Hiessl and Skrandies, 2013; Lui et al., 2020; Paterlini et al., 2021; Sobolev et al., 2021). The research conducted by Osgood et al. (1957), on different objects of investigation and among culturally different samples, showed that the SD method can highlight “latent cognitive structures” commonly referring to three different dimensions, mutually independent. These dimensions resulted stable and transversal in most factor analyses on SD instruments, even if different authors have given specific names to the factors according to the specific sample and object of study (e.g., Salcuni et al., 2007; Ma et al., 2018). Each dimension corresponds to an attributive psychological factor, which collectively represent the subjective attitude toward the object under investigation. The three general widespread latent dimensions are as: (1) Evaluation (indicates the positivity/negativity of the evaluated element): It is measured through the use of pairs of adjectives, such as “good – bad,” “nice – ugly,” and “pleasant – unpleasant”; (2) Potency (indicates the strength/weakness of the evaluated element): It is measured through pairs of adjectives, such as “strong – weak,” “large – small,” and “heavy – light”; and (3) Activity (indicates the activity/passivity of the item evaluated): It is measured through pairs of adjectives, such as “active – passive” and “fast – slow.” The greater proportion of variance in factorial analyses relates to the Evaluation dimension, which has been considered by Osgood and collaborators the one that most concretizes the concept of attitude (1957).

**Figure 1:**
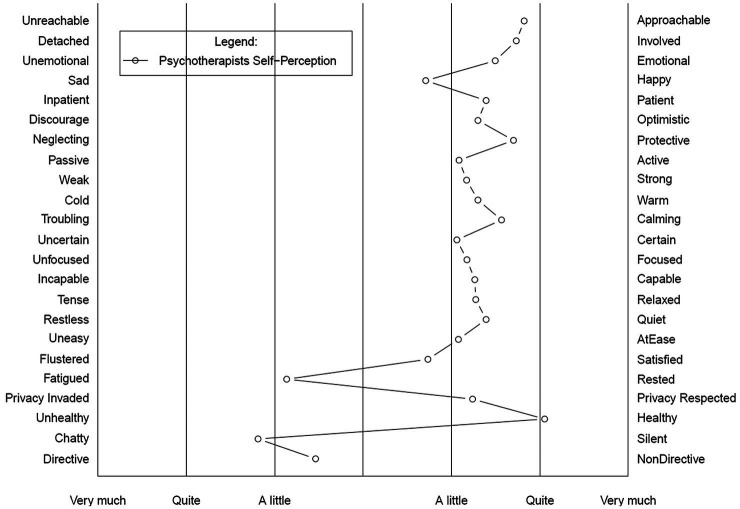
Psychotherapists’ self-perceptions when conducting telepsychotherapy during the pandemic – semantic differential plot.

In the current study, the SD scale was composed of 23 pairs of adjectives with reverse or polar meaning (Figure 1), selected *ad-hoc* by the authors. Respondents rated each pair on a 7-point scale (1=negative pole; 7=positive pole). Table 1 provides an overview of the SD bipolar items (translated into English).

**Table 1:** Semantic differential items and factor saturation.

Item	AA	AP	WB	I	
Approachable–Unreachable	0.73				*R*
Involved–Detached	0.71				*R*
Emotional–Unemotional	0.62				*R*
Happy–Sad	0.54				*R*
Patient–Impatient	0.54				*R*
Optimistic–Discouraged	0.50				*R*
Privacy Respected–Privacy Invaded	0.48				*R*
Passive–Active		0.73			
Weak–Strong		0.72			
Cold–Warm		0.64			
Troubling–Calming		0.58			
Uncertain–Certain		0.56			
Unfocused–Focused		0.47			
Incapable–Capable		0.45			
Protective–Neglecting		0.43			*R*
Frustrated–Satisfied		0.38			
Relaxed–Tense			0.68		*R*
Quiet–Restless			0.59		*R*
At ease–Uneasy			0.47		*R*
Fatigued–Rested			0.38		
Healthy–Unhealthy			0.34		*R*
Directive–Non-directive				0.62	
Chatty–Silent				0.44	
Eigenvalue	3.83	3.29	2.26	0*.98*	
Cronbach’s alpha	0.80	0.87	0.73	0.50	

AA, Affective Availability (factor 1); AP, Attitude Predisposition (factor 2); WB, Well Being (factor 3); I, Interventionism (factor 4); and R, reversed item.

### Sample Description

Participants were 281 Italian psychotherapists aged 28–76years (*M*_age_=45.15; *SD*=10.2), of whom most were female (*N*=235; 83.6%). Data were collected homogeneously from all parts of Italy (north: 37%; central: 35%; south: 28%). Most participants had a psychoanalytical theoretical orientation (60.9%); the remainder was cognitive–behavioral (15.3%), systemic (8.5%), humanistic (12.8%), and integrated (2.5%). The sample reported a mean clinical experience of 13years (*SD*=8.46; range: 1–40), whereas their experience with online psychotherapy was consistently shorter. Specifically, 43.8% had never practiced online psychotherapy prior to the pandemic, 42.7% had only practiced it occasionally, and 13.5% had practiced it frequently. Finally, 60% of participants practiced individual psychotherapy, 10% practiced both individual and group psychotherapy, and 40.9% practiced psychotherapy in multiple client settings (i.e., individual, group, couple, and family).

### Data Analysis

All analyses were performed using the R software (v. 4.0.3). After assessing the demographic characteristics of the sample, the data analysis focused primarily on the SD items, for which an inventory plot was drafted to evaluate the overall sample trends with respect to attitudes and affective states. Subsequently, the R “*psych*” package (Revelle, 2019) was used to conduct a factor analysis with the principal components method and oblique Promax rotation.

The analysis was conducted after Bartlett’s test of sphericity was performed to test the null hypothesis that the correlation matrix was an identity matrix. To determine the number of factors that should be retained, Horn’s parallel analysis was performed with an eigenvalue > 1. To detect the variables to be included in each factor, a cutoff loading value > 0.3 (after Promax rotation) was used, as in previous studies (e.g., Parker et al., 2011; Koelewijn et al., 2014; Fagioli et al., 2015; Barnes and Mongrain, 2020). The resulting items in each factor were scored as means.

Finally, following a top-down approach, characteristics of the psychotherapists falling into the “Positive” (scores on each factor>4) and “Negative” (scores on each factor<4) poles of the SD factors were examined and compared using a chi-squared test with Yates’s correction (*p*<0.05). A Monte Carlo test for significance testing (Hope, 1968) with *B*=2500 replicates was also applied, as it enables more reliable results to be obtained when chi-squared test assumptions are not fully satisfied. Lastly, the proportion of clients treated via telephone or video call and the client dropout rate was expressed as percentages. These variables were thus operationalized as categories representing decimal ranges to facilitate the chi-squared test.

## Results

### Psychotherapists’ Self-Perceptions

Figure 1 reports the overall sample’s self-perception profile, referring to the psychotherapists’ feelings when conducting clinical activity online during the emergency period. A generally favorable trend emerged as: on average, the psychotherapists reported an affective state and attitude closer to the positive pole. They also reported feeling quite confident and competent in their clinical practice. Nonetheless, they tended to feel more fatigued than they had prior to the pandemic, as well as more talkative and directive during telematic sessions.

### Factor Analysis

A factor analysis using the principal component method was conducted to identify the factorial structure of the SD items. Bartlett’s test of sphericity was initially performed, rejecting the null hypothesis that the correlation matrix was an identity matrix (*X*^2^=2600.66; *df*=253; *p<0*.001). The results of the factor analysis suggested a four-factor structure (Table 1). The first factor, “Affective Availability” (AA), accounted for 17% of the total variance on seven items and is in line with the Evaluation dimension described by Osgood et al. (1957); the second factor, “Attitude Predisposition” (AP), accounted for 14% of the variance and overlaps on the Osgood’s Activity dimension; and the third factor, “Well-being” (WB), accounted for 10% of the variance and substantially coincides with the Osgood’s Potency dimension. All three factors properly described the psychotherapists’ attitudes and affective states, with an eigenvalue ≥1. Differently, the fourth and final factor only accounted for 4% of the variance and showed an eigenvalue slightly below the cutoff (i.e., eigenvalue < 1). This factor better described the psychotherapists’ behavioral predispositions and was therefore named “Interventionism” (I). As such, only the first three factors were used to define the Positive and Negative self-perception profiles. The four-factor structure showed satisfactory fit indices (*X*^2^ = 173.83; *p*=0.34; RMSEA=0.05; TLI=0.93).

The overall sample’s mean scores and standard deviations for the four factors were as follows: Affective Availability, *M*=5.40, *SD*=0.985; Attitudinal Predisposition, *M*=5.23, *SD*=1.001; Wellbeing, *M*=4.99, *SD*=1.09; and Interventionism, *M*=3.14, *SD*=1.05. When participants at the extremes (i.e., Positive self-perception profile=scores > 4; Negative self-perception profile=scores < 4) of the Affective Availability, Attitudinal Predisposition, and Wellbeing factors were extracted from the analysis, the sample resulted as quite homogeneous, showing a mainly positive self-perception during telematic sessions. Specifically, 193 participants (68.63%) fell within the Positive self-perception profile, while only 11 (3.92%) fell within the Negative self-perception profile. Psychotherapists with a Positive profile were aged 28–72years (*M*=45.02; *SD*=10.14; 85.5% female) and had a mean experience as a licensed psychotherapist of 12.93years (*SD*=8.34). Differently, psychotherapists with a Negative profile were aged 33–66years (*M*=48; *SD*=10.05; 54.5% females) and had a mean experience as a licensed psychotherapist of 15.73years (*SD*=8.17). With respect to Interventionism, both profiles appeared similar: The mean level of Interventionism for the Positive self-perception profile was 3.1 (*SD*=1.12), while for the Negative self-perception profile it was 3.32 (*SD*=0.51).

The characteristics and item response frequency (Table 2) of both profiles were compared using a chi-squared test with Yates’s correction. A Monte Carlo significance test (*B*=2500) was also performed. The results showed that the profiles significantly differed with respect to their gender distribution (*X*^2^_(2)_=7.81; *p*= 0.02), overall satisfaction with telepsychotherapy (*X*^2^_(1)_=3.80; *p*= 0.05), number of therapeutic sessions conducted via telephone (*X*^2^_(9)_=16.82; *p*= 0.05), and perception of clients’ ability to maintain privacy during sessions (*X*^2^_(4)_=19.59; *p<* 0.01). Following the Monte Carlo test, differences between the profiles were supported only with respect to overall satisfaction with telepsychotherapy (*X*^2^_(1)_=5.20; *p*= 0.04) and the perception of clients’ ability to maintain privacy during sessions (*X*^2^_(5)_=19.59; *p*= 0.04). In particular, compared to the Negative self-perception profile, the Positive profile was characterized by greater satisfaction with the new working modality and a greater perception that clients could maintain privacy during the telematic sessions. Differently, a greater percentage of psychotherapists with a Negative profile reported that they never perceived that patients could maintain privacy during the online sessions. No differences in dropout rates between the two profiles have emerged.

**Table 2:** Differences between positive and negative self-experience profiles.

	Positive profile	Negative profile
**Work context**		
Independent practice	58.5%	54.5%
Public mental health service	9.3%	18.2%
Independent practice and public service	32.1%	27.3%
**Setting**		
Individual	46.6%	54.5%
Individual and group	8.8%	9.1%
Individual and couple/familial	36.3%	9.1%
Individual and group and couple/familial	8.3%	27.3%
**Theoretical Orientation**		
Psychoanalytical	56.6%	81.8%
Cognitive–behavioral	17.1%	0
Systemic	9.8%	9.1%
Humanistic	13.5%	9.1%
Integrated	3.1%	0
**Previous experience with telematic psychotherapy**		
Never	44%	18.25
Rarely	40%	72.7%
Often	15.5%	9.1%
**Perceived patients’ difficulty maintaining privacy** ^*^		
Never	**21.8%**	**9.1%**
Rarely	**0.5%**	**45.5%**
Often	**56%**	**9.1%**
Always	**21.8%**	**36.4%**
*Theoretical compatibility with online psychotherapy* ^#^	83.4%	63.6%
*Satisfaction with telematic psychotherapy* ^#^ ^*^	**69.4%**	**36.4%**
*Therapy sessions via video call* ^§^	53.8%	40.45%
*Therapy sessions via telephone* ^§^	16.1%	18.64%
*Dropout rate* ^§^	40%	43.82%

* Significant difference after applying Monte Carlo test procedure;

# Dycotomic variable;

§ mean of the percentages of patients treated through phone calls or video calls and of dropouts (see Data analysis section for their operationalized as categories to perform analysis). Bold values indicate the levels in which the two profiles (i.e, positive and negative) were significantly different from each other.

## Discussion

The current study represents an important contribution to the literature with respect to the connection between psychotherapists’ attitudes, subjective experiences, and emotional states related to telepsychotherapy and their overall satisfaction with the telematic treatment modality. The findings are fundamental, because previous research has documented that therapists’ subjective characteristics are complexly associated with relational and technical factors that relate to psychotherapeutic treatment outcome (Sarracino et al., 2013; Heinonen et al., 2014; Lingiardi et al., 2018; Heinonen and Nissen-Lie, 2020).

The results, referring to a large sample of psychotherapists in Italy, suggest that Italian psychotherapists were able to rapidly and flexibly adapt to the suddenly imposed working modality of telepsychotherapy during the COVID-19 pandemic. Participants, on average, reported a positive self-perception during telepsychotherapy sessions, in alignment with recent evidence of mental health professionals’ good acceptance of online clinical interventions during the pandemic (Machluf et al., 2021). The Italian psychotherapists involved in the current study also reported feeling quite confident and competent in their online clinical practice; this represents a more encouraging finding than those reported by Aafjes-van Doorn et al. (2020).

The current results are additionally promising since telepsychotherapy – especially when considered outside the emergency context, when in-person contact is more accessible – involves advantages and drawbacks for both psychotherapists and their clients. International studies have demonstrated a fairly unanimous consensus that the main benefit of online treatment is the accessibility it provides to distant or remote clients, including those who are homebound due to serious medical and/or social conditions and those who are unable to easily travel (Gordon et al., 2015; Apolinário-Hagen et al., 2017). More than a decade ago, a review by Simpson (2009) identified the reduced financial and time costs made possible with online psychotherapy as a key asset of this treatment modality. Furthermore, the reduction of psychotherapeutic waiting lists has also been identified as an advantage of online interventions (Apolinário-Hagen et al., 2017).

However, in line with previous investigations (Békés and van Doorn, 2020), the current study found that psychotherapists felt more fatigued when administering online treatment. This fatigue could be identified as a drawback of telepsychotherapy, potentially associated with psychotherapists’ limited access to non-verbal (including emotional) cues during online sessions (Chherawala and Gill, 2020), demanding greater effort to capture all relevant background information for clinical practice. However, certain precautions may circumvent this problem, including the use of a wider webcam framing, which would allow clients and psychotherapists to visually “share” a larger portion of their bodies and surroundings (Grondin et al., 2020). Moreover, a systematic review of the literature on videoconferencing in psychotherapy concluded that participants were generally able to communicate and decode emotions accurately (Backhaus et al., 2012). Importantly, some warnings have emerged from the affective neuroscience field, as Schiano Lomoriello et al. (2018) demonstrated that the perceived physical distance between two interacting individuals could modulates the empathic reaction between them. Nonetheless, considering the elevated level of distress caused by the COVID-19 outbreak on the general public and mental health professionals worldwide, it is reasonable to hypothesize that the greater fatigue perceived by psychotherapists practicing online during the pandemic could be ascribed not only to the telematic modality, but also to the taxing external circumstances (Cao et al., 2020; Castellini et al., 2021).

Moreover, this study also underlines how the online modality may have changed psychotherapists’ interactive styles during sessions. Specifically, the clinicians reported that they were much more conversational and directive during telematic sessions. This could be interpreted as an attempt to compensate for physical distance from clients and/or a concern that clients may perceive them as less connected (Békés and van Doorn, 2020); it may also represent an effort to be more involved in dialog with clients, as this is a well-known factor in determining good treatment outcomes (Orlinksy and Howard, 1986). However, increased verbal expressivity on the therapist’s part may have important implications for treatment. This may be particularly true for psychoanalytic therapists, for whom a neutral and fairly structured therapeutic setting is a technical tenet essential to accomplish different clinical objectives, such as favoring patients’ regression and emotional processing (Winnicott, 1964; Stern, 2002). Although systematic research on the effect of therapist’s talkativeness on treatment outcome is somewhat limited, previous studies suggested that successful therapists tend to talk less than their clients and that, especially with patients who have undergone the most improvement, therapists are less loquacious (Hölzer et al., 1996). On the other hand, less talkative clinicians can be perceived by their clients as aloof and emotionally disengaged, with problematic consequences for treatment effectiveness (Lane et al., 2002). Furthermore, the therapist’s increased verbal activity during telepsychotherapy could also be interpreted in the light of the theory and research regarding common therapeutic factors. Specifically, while abstinence and therapeutic use of silence are well-known techniques with theoretical and empirical justification from the psychoanalytical framework (Hölzer et al., 1996; Stern, 2002), decades of research in psychotherapy attest to the relevance of non-specific factors for treatment outcome (Luborsky, 1975; Wampold, 2015). Among others, the therapist’s empathic attitude during sessions and the therapeutic alliance is established as key ingredients of successful treatment (Luborsky, 1995; Ribeiro et al., 2013). Considering also recent evidence that clients tend to be more satisfied and less worried about potential difficulties of telepsychotherapy than therapists (Etzelmueller et al., 2018), it seems reasonable that clinician’s enhanced talkativeness might represent an emotionally attuned response toward the patient who might actually need more active engagement, reassurance, and containment, particularly in difficult times as the pandemic.

Moreover, by conducting a factorial analysis of the SD scale, we identified four latent dimensions describing psychotherapists self-perception during telepsychotherapy. Based on these latent variables, we identified two broad profiles, i.e., Positive and Negative self-perception profiles, which are related to psychotherapists’ affective and attitudinal states toward telepsychotherapy. The positive self-perception profile would describe psychotherapists viewing themselves as more engaging and available (i.e., higher scores on the Affective Availability factor) as well as more present and attuned (i.e., higher scores on the Attitudinal Predisposition factor) in interacting with patients during telematic sessions. On the contrary, the Negative self-perception profile would describe psychotherapists perceiving a sense of detachment and reduced self-efficacy, as well as a state of uneasiness and agitation signaling an overall deflation of personal strength (i.e., lower score on the Wellbeing factor).

Noteworthy, compelling differences emerged by comparing the Positive and the Negative self-perception profiles regarding psychotherapists’ characteristics and clinical practices. Specifically, psychotherapists that fall into the Positive self-perception profile were more satisfied with the online therapeutic modality and more likely to perceive that their clients were able to maintain privacy during sessions. No significant differences emerged between the two profiles regarding the clinicians’ theoretical orientation. However, more research is needed to establish whether some theoretical orientation in psychotherapy can be more suitable for the online setting than others. Taken together, these findings support the link between therapists’ attitudes toward treatment, sense of ease during sessions, and overall perceived effectiveness.

Despite the importance of the findings, it is worthwhile to recognize some limitations of the study. Specifically, the cross-sectional nature of the study did not allow for causal inferences. Moreover, the reliability of the reported differences was limited by the uneven sample distribution between profiles; however, the Monte Carlo test for significance provided meaningful support for the reliability of the findings. Another limitation of the study can be due to the sample characteristics; in particular, many psychotherapists included in the sample reported to be psychoanalyst working in a private setting, making more difficult to generalize the results. Moreover, due to the nature of data collected, we cannot exclude that there might be difference due to the patients. Particularly, psychotherapists may have had different experience with those patients who started psychotherapy after the beginning of the lockdown compared to those who were already in treatment. Nevertheless, it is important to note that the data collection lasted 1month, starting approximately 4weeks after the Government’s first restriction. Consequently, we hypothesize that patients who started psychotherapy during this period, if any, should be a tiny proportion.

In conclusion, the current study highlighted a quite positive experience of Italian psychotherapists in their provision of telepsychotherapy during the early stages of the COVID-19 outbreak in Italy. However, personal difficulties, such as fatigue, emerged, as well as a greater conversational and directive attitude during sessions. These results, together with related findings reported in the literature (e.g., Békés and van Doorn, 2020), may stimulate further investigations to inform the development of educational programs for professionals interested in this new modality. Moreover, in light of recent evidence showing European clinicians’ more negative attitudes toward online treatment compared to their North American and Canadian counterparts (Aafjes-van Doorn et al., 2020), further research into European clinicians’ personal experiences related to telepsychotherapy may be particularly relevant. Given the interrelationship of attitudes, experience, and treatment efficacy (Reese et al., 2016; Tonn et al., 2017; Aafjes-van Doorn et al., 2020), the professional community of European clinicians may be at greater risk of falling short in meeting the increased needs for – and exploiting the potential opportunities of – online mental care. In this regard, the pandemic era, when many healthcare professionals have had to revert to online psychotherapy or intensify its use, represents a particularly crucial time, because therapists’ experiences may shape their views about this treatment modality and impact their attitudes toward future online work. Notably, the journalistic communication in Italy offered a contrasting position to interpret the pandemic; as a transient “unexpected event” (i.e., “emergency situation”) and an “attempt of routinization” of the related collective changes in the population (Papapicco, 2020). This aspect is relevant since how people refer to pandemics could intensify the feeling of information uncertainty and implements the construction of different social representations about the current scenario. Future follow-up studies are needed to address these aspects, which might play a role in defining the future of telepsychotherapy.

## Data Availability Statement

The raw data supporting the conclusions of this article will be made available by the authors, without undue reservation.

## Ethics Statement

The studies involving human participants were reviewed and approved by Comitato Etico per la Ricerca Psicologica (Area 17). The patients/participants provided their written informed consent to participate in this study.

## Author Contributions

TB and ASL developed the survey. EM analyzed the data. EM and EG wrote the first draft of the manuscript. SS conceived the research study and contributed to the development of the survey. SS, TB, VL, and ASL contributed to the interpretation of the results and critically reviewed the final draft of the manuscript. All authors contributed to the article and approved the submitted version.

## Conflict of Interest

The authors declare that the research was conducted in the absence of any commercial or financial relationships that could be construed as a potential conflict of interest.

## Publisher’s Note

All claims expressed in this article are solely those of the authors and do not necessarily represent those of their affiliated organizations, or those of the publisher, the editors and the reviewers. Any product that may be evaluated in this article, or claim that may be made by its manufacturer, is not guaranteed or endorsed by the publisher.
